# Molecular Characterization of Watermelon Chlorotic Stunt Virus (WmCSV) from Palestine

**DOI:** 10.3390/v6062444

**Published:** 2014-06-20

**Authors:** Mohammed S. Ali-Shtayeh, Rana M. Jamous, Omar B. Mallah, Salam Y. Abu-Zeitoun

**Affiliations:** Biodiversity and Biotechnology Research Unit, Biodiversity and Environmental Research Center-BERC, Til, Nablus 970, Palestine; E-Mails: rana@berc.ps (R.M.J.); omarm@berc.ps (O.B.M.); salamaz@berc.ps (S.Y.A.-Z.)

**Keywords:** WmCSV, SLCV, *Begomovirus*, watermelon, Palestine, RCA

## Abstract

The incidence of watermelon chlorotic stunt disease and molecular characterization of the Palestinian isolate of Watermelon chlorotic stunt virus (WmCSV-[PAL]) are described in this study. Symptomatic leaf samples obtained from watermelon *Citrullus lanatus* (Thunb.), and cucumber (*Cucumis sativus* L.) plants were tested for WmCSV-[PAL] infection by polymerase chain reaction (PCR) and Rolling Circle Amplification (RCA). Disease incidence ranged between 25%–98% in watermelon fields in the studied area, 77% of leaf samples collected from Jenin were found to be mixed infected with WmCSV-[PAL] and SLCV. The full-length DNA-A and DNA-B genomes of WmCSV-[PAL] were amplified and sequenced, and the sequences were deposited in the GenBank. Sequence analysis of virus genomes showed that DNA-A and DNA-B had 97.6%–99.42% and 93.16%–98.26% nucleotide identity with other virus isolates in the region, respectively. Sequence analysis also revealed that the Palestinian isolate of WmCSV shared the highest nucleotide identity with an isolate from Israel suggesting that the virus was introduced to Palestine from Israel.

## 1. Introduction

The family *Geminiviridae* comprises small circular single-stranded DNA viruses that cause severe diseases in major crop plants worldwide. On the basis of genome organization, host range, and type of insect vector, the family is divided into seven genera: *Becurtovirus*, *Begomovirus Curtovirus*, *Eragrovirus*, *Mastrevirus*, *Topocuvirus*, and *Turncurtovirus* [1,2]. Members of the largest genus, *Begomovirus* (192 species), infect primarily dicotyledonous plants and are transmitted by the whitefly *Bemisia tabaci* [3]. Many begomoviruses have a bipartite genome (two DNAs of approximately 2600 nt each, termed DNA-A and DNA-B); a DNA-A component encoding all the protein functions necessary for virus replication in a single cell, while the DNA-B component provides movement functions required for systemic spread. Begomoviruses are transmitted in a persistent way by the whitefly *Bemisia tabaci* (Gennadius). They negatively affect important crops such as cucurbits, legumes, cotton, ornamentals, peppers, and tomato [4,5,6].

Bipartite begomoviruses usually contain 7 open reading frames (ORF). DNA-A contains five ORFs, which encode for proteins involved in replication, regulation of gene expression, and encapsidation, while DNA-B contains two ORFs, encoding for proteins involved in viral movement and symptom development [7,8]. Begomoviruses have spread rapidly among cucurbit crops throughout the world, and such invasions were caused by *Squash leaf curl virus* (SLCV) [6,9,10,11], *Cucurbit leaf curl virus* (CuLCV) [12], *Melon chlorotic leaf curl virus* (MCLCuV) [13,14] and *Watermelon chlorotic stunt virus* (WmCSV) [6,15,16,17,18].

In summer and fall of the years 2008 and 2009, a new disease was detected on cucumber plants grown in 6 greenhouses and 26 fields in 9 localities in Tulkarm area in Palestine. Infected cucumber plants exhibited vein yellowing, mottling, stunting of young leaves, and reduction of yield. The disease was associated with elevated whitefly population. However, in summer of 2010, the disease reappeared on watermelon plants, total DNA was extracted from the infected symptomatic plants and used as template for PCR amplification, using the universal begomovirus degenerate primer pair PAL1v1978 and PAR1c496 for DNA A [19]. The specific begomovirus band was indeed amplified and, following sequencing, it was determined that the new watermelon disease was induced by WmCSV [20].

WmCSV was first identified in Yemen, and then in Sudan [21,22,23]. Outside of the Red Sea region, the virus has been found in Iran [15,17], later on, between the years 2010–2012, the virus was reported in Israel, Jordan, Lebanon and Oman [6,16,24,25]. The complete genomes of two WmCSV isolates, from Sudan and from Iran, have been cloned and sequenced [15]. The sequence of a third isolate, from Yemen, is also available in GenBank (accession numbers AJ012081 and AJ012082), other isolates from Israel, Jordan, Lebanon and Oman are now available in the GenBank. The virus infects nearly all cultivated cucurbits, but induces severe damage mainly to watermelon and to melon (*Cucumis melo* L.) plants [6].

The objective of this work is to characterize the Palestinian isolates of WmCSV at the molecular level.

## 2. Results and Discussion

### 2.1. Field Survey

**Figure 1:**
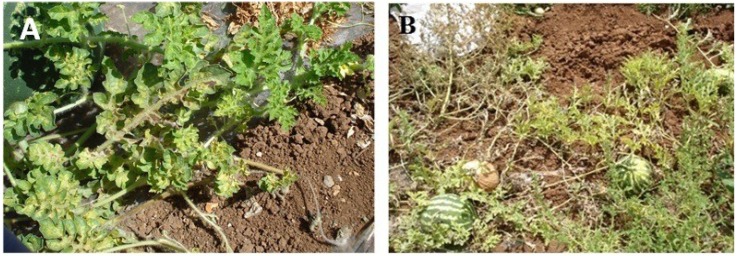
Severe leaf curling (**a**) and chlorotic mottling (**b**) observed on watermelon infected with WmCSV.

**Figure 2:**
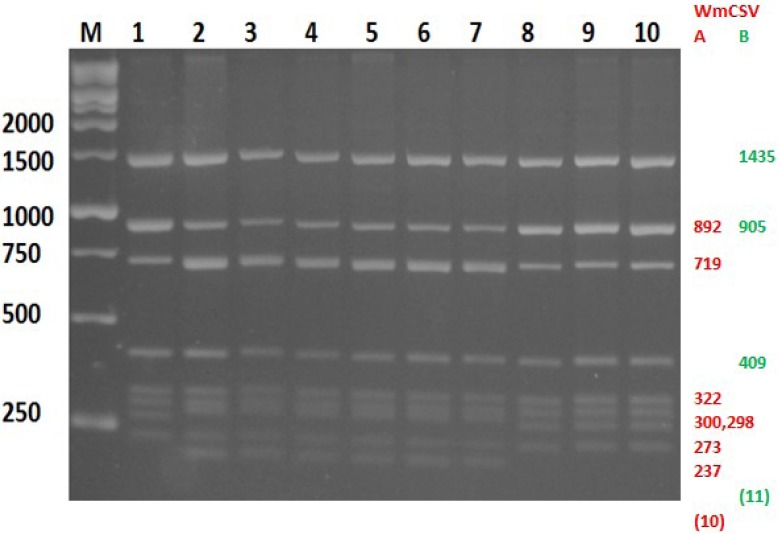
Gel electrophoresis of rolling circle amplification (RCA) followed by restriction fragment length polymorphism (RFLP) products using *Hpa* II. The expected fragments sizes (nts) for DNA-A (red) and DNA-B (green) shown obtained by *in silico* digestion of the sequenced WmCSV Palestinian isolate is shown at the right side of the gel. Values in brackets refer to bands that are too small to be resolved in this gel system. M, molecular weight marker; 1–10, watermelon samples infected with WmCSV.

In 2010, 2011 and 2013 leaf samples were collected from 481 watermelon and 20 cucumber plants that showed disease symptoms similar to those previously described to be caused by WmCSV. Infected plants showed symptoms like yellow veins, mottling, and severe stunting of young leaves (Figure 1). Disease incidence (number of symptomatic plants/50 plants) reached 98% in some of the 23 watermelon and cucumber fields surveyed. This may partially explain the reported decline of watermelon-cultivated areas in Palestine from 400 ha in 2007 to 111 ha in 2011 [26]. Analysis of the collected samples by PCR using degenerate primers revealed that 81.4% (408 out of 501) of the samples were infected with geminiviruses (Table 1). In samples collected in 2010, 53 watermelon samples out of 152 samples collected from Jenin and Qalqilia, which were positive with degenerate primers, were shown to be infected with WmCSV-[PAL] by PCR using specific primers. However, symptomatic leaf samples which were negative with specific primers (99 samples) and 47 samples (37 from Jenin, and 10 from Qalqilia) which have been shown to be positive with specific primers in parallel with a DNA sample of standard WmCSV (KC462552) were analyzed for WmCSV by RCA/RFLP analysis. All samples produced an RCA product, and 73 showed an RFLP digestion pattern similar to the positive control and *in silico* digestion of WmCSV [27] (Figure 2), while the rest of the samples (79) gave a different pattern similar to SLCV digestion pattern. ELISA test using virus-specific antibodies [28,29,30] and polymerase chain reaction (PCR) using either specific or degenerated primers [31,32,33] were the ordinary methods used for detection of geminiviral infection in plant samples. However, RCA combined with RFLP improved the diagnosis of geminiviral infection and was shown to be a suitable and more sensitive tool (10 ng of DNA sample would be enough using RCA) for geminiviral infection screening [27,34,35,36,37] due to the several advantages over other methods. The diagnosis of geminiviral infection by using RCA/RFLP is largely independent of source plant type and origin, viral genome organization or sample preparation method, since neither specific primers nor expensive equipment, like a thermocycler are needed for the reaction [38]. On the other hand, some factors might inhibit the PCR reaction which might explain the samples which were positive with RCA and negative with PCR using specific primers. Leaf samples collected from Jenin and Qalqilia were found to be mixed infected with WmCSV-[PAL] and SLCV-[PAL] (48%). Mixed infections with two or three whitefly transmitted viruses were also very common in cucurbits fields in Lebanon and Jordan [16,24]. Moreover, recent results have demonstrated that co-infection of melon plants with both WmCSV and SLCV results in a synergistic reaction characterized by severe symptoms and a major yield reduction [6,39]. Hence, these two viruses, WmCSV and SLCV, pose a danger to cucurbit crops around the Mediterranean. On the other hand, watermelon and cucumber samples collected in 2010, 2011 and 2013 from Tulkarm, Jenin and Jericho respectively, which were positive in degenerate primers, and negative with WmCSV specific primers were shown to be positive by PCR for SLCV using specific primers (infection rate ranged between 50%–100%). SLCV was reported for the first time in Palestine in 2008 [39]; very recently the disease was shown to threaten cucurbits production in Palestine, and the incidence rate was reported to exceed 90% on squash plants in the Qalqilia district [11].

**Table 1:** Detection of Watermelon chlorotic stunt virus-[PAL] in watermelon and cucumber fields in Palestine during 2010–2013 using Polymerase Chain Reaction (PCR) and Rolling Circle Amplification (RCA).

Season/Year	Area	Crop	Fields	+ve by deg. primers *	+ve for WmCSV (PCR)	+ve for WmCSV (RCA)	Total no. of samples +ve for WmCSV (%)	No. of Samples mixed Infected with SLCV (% ^ϕ^)
Summer/2010	Tulkarm	Watermelon	3	58/62	0	0	0 (0)	50 (80.6)
		Cucumber	1	19/20	0	0	0 (0)	19 (95)
	Jenin	Watermelon	4	65/110	43	37	43 (39.1)	27 (62.7)
	Qalqilia	Watermelon	4	87/105	10	36	36 (34.3)	11(30.5)
Summer/2011	Jenin	Watermelon	4	ND (120)	0	0	0 (0)	100 (83.3)
Arifa S. Khan	Jericho	Watermelon	7	59/84	0	0	0 (0)	54 (64.3)
Total			23	288/501	53	73	79 (15.8)	261 (52.1)

* Samples which were positive (+ve) with degenerate primers and negative for WmCSV specific primers have shown to be positive for SLCV using SLCV specific primers; ND, not done; ^ϕ^ % calculated from total number of samples infected with WmCSV.

Watermelon chlorotic stunt disease caused by WmCSV has progressed to alarming levels in many eastern Mediterranean countries; it was identified for the first time in Israel in 2004 [6]. Recently, the virus was detected for the first time in south Lebanon on cucumber, melon, watermelon, and squash plants [24], while it was detected on melon and watermelon plants in the southern part of Jordan Valley, and the eastern part of Jordan [16]. SLCV and WmCSV, have been reported in many eastern Mediterranean countries [6,10,11,24,40,41].

### 2.2. Cloning and Sequencing of WmCSV-[PAL]

The full-length DNA-A from 9 isolates and DNA-B from 1 isolate of WmCSV-[PAL] were amplified by PCR from symptomatic watermelon plants collected from Jenin and Qalqilia using virus-specific primers and RCA product as a template (Table 2). Amplified PCR products were cloned and sequenced, and sequences of the Palestinian isolates of WmCSV-[PA:Pal:10] were deposited in the GenBank under accession numbers listed in Table 3.

**Table 2:** Oligonucleotide primers used to amplify DNA-A and DNA-B of WmCSV-[PA:Pal:10] by polymerase chain reaction.

Primer Name	5'–3' Sequence	Target	Fragment Size
WmA150F ^1^	GTCAGTATGTGGGATCCATTGC	DNA-A	1201 bp
WmA 1350R ^1^	GCAAATACGATTCAACCACAACC		
WmA170R ^1^	GCAATGGATCCCACATACTGAC	DNA-A	1597 bp
WmA1325F ^1^	GGTTGTGGTTGAATCGTATTTGC		
WmB672F	CGCCGTTGCCTGGAGGATGTTCAC	DNA-B	1329 bp
WmB2000R	GCAGCACAGGCTGCCTTCACCTTC		
WmB1977F	GAAGGTGAAGGCAGCCTGTGCTGC	DNA-B	1479 bp
WmB695R	GTGAACATCCTCCAGGCAACGGCG		

^1^ Primers sequences were kindly provided by Dr. Moshe Lapidot (Agricultural Research Organization, Beit Dagan, Israel).

**Table 3:** Origins of WmCSV-[PA:Pal:10] isolates and features of the DNA-A and DNA-B components.

		DNA-A								DNA-B			
			Position of Genes (Coordinates of Start/Stop Codons [Predicted Coding Capacity in kDa]									Position of Genes (Coordinates of Start/Stop Codons [Predicted Coding Capacity in kDa]	
			AV2	AV1	AC1	AC2	AC3	AC4	AC5			BC1	BV1
Isolate	Accession Number	Size (nt)	nt	nt	Nt	nt	Nt	nt	nt	Accession Number	Size (nt)	nt	nt
PAL-1-J40	KC462552	2751	154–513 (13.2)	314–1090 (28.7)	1539–2624 (40.2)	1234–1641 (15.1)	1087–1491 (14.9)	2327–2470 (5.3)	218–985 (28.4)	KC462553	2760	1326–2321 (36.5)	522–1286 (28.3)
PAL-2-J44	KJ854912	2753	156–515 (13.2)	316–1092 (28.7)	1541–2626 (40.2)	1234–1641 (15.1)	1089–1493 (14.9)	2329–2472 (5.3)	220–987 (28.4)			-	-
PAL-3-J45	KJ854913	2752	155–514 (13.2)	315–1091 (28.7)	1540–2625 (40.2)	1233–1640 (15.1)	1088–1492 (14.9)	2328–2471 (5.3)	219–986 (28.4)			-	-
PAL-4-J56	KJ854914	2752	155–514 (13.2)	315–1091 (28.7)	1540–2625 (40.2)	1233–1640 (15.1)	1088–1492 (14.9)	2328–2471 (5.3)	219–986 (28.4)			-	-
PAL-5-J67	KJ854915	2752	155–514 (13.2)	315–1091 (28.7)	1540–2625 (40.2)	1233–1640 (15.1)	1088–1492 (14.9)	2328–2471 (5.3)	219–986 (28.4)			-	-
PAL-6-Q13	KJ854916	2753	156–515 (13.2)	316–1092 (28.7)	1541–2626 (40.2)	1234–1641 (15.1)	1089–1493 (14.9)	2329–2472 (5.3)	220–987 (28.4)			-	-
PAL-7-Q17	KJ854917	2752	156–515 (13.2)	316–1092 (28.7)	1541–2626 (40.2)	1233–1640 (15.1)	1088–1492 (14.9)	2328–1471 (5.3)	219–986 (28.4)			-	-
PAL-8-Q20	KJ854918	2753	156–515 (13.2)	316–1092 (28.7)	1541–2626 (40.2)	1234–1641 (15.1)	1089–1493 (14.9)	2329–2472 (5.3)	220–987 (28.4)			-	-
PAL-9-Q23	KJ854919	2753	156–515 (13.2)	316–1092 (28.7)	1541–2626 (40.2)	1234–1641 (15.1)	1089–1493 (14.9)	2329–2472 (5.3)	220–987 (28.4)			-	-

### 2.3. Sequence Analysis of DNA-A and DNA-B of WmCSV-[PA:Pal:10]

**Figure 3:**
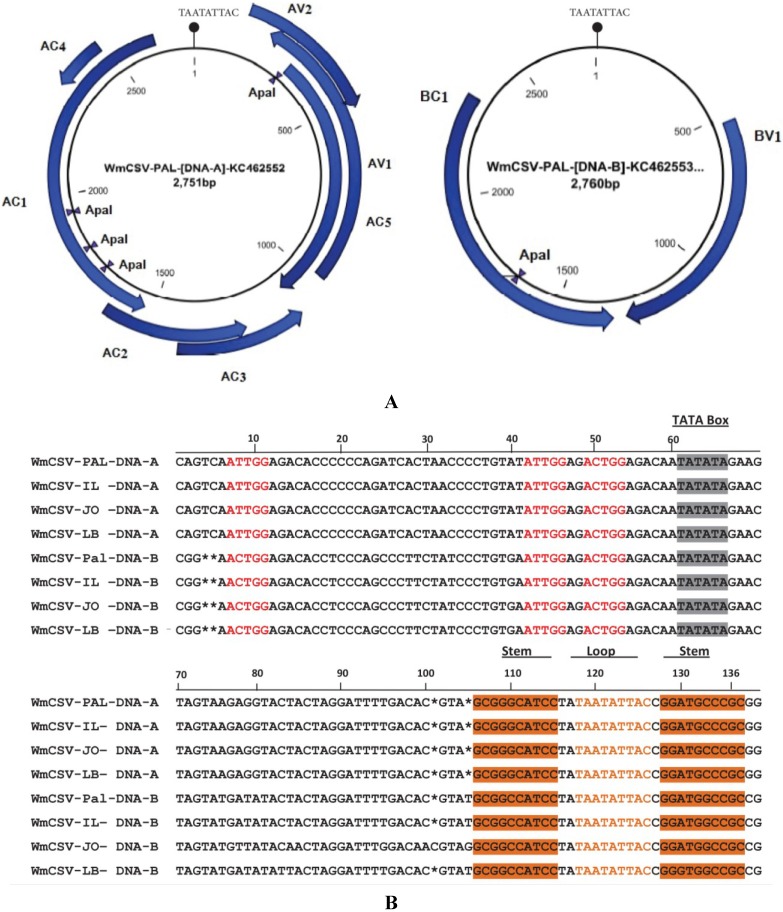
Arrangement of the genomic components of WmCSV-[PA:Pal:10] (**A**). Alignment of Common Region sequences of the DNA-A and DNA-B components of WmCSV-[PA:Pal:10] and the database sequences from neighboring countries (Jordan, Lebanon and Israel) (**B**). Spaces (*) were introduced to optimize the alignment. Stem-loop sequences (orange highlighting and orange text respectively) of predicted stem-loop structure, position of introns (red text) and TATA box (highlighted) of the Rep promoter are indicated.

Sequence analysis using the CLC Main Workbench 6.9 [42] showed that DNA-A of WmCSV-[PA:Pal:10] shared high nucleotide identity (99%–100%) with other isolates (Table 3), indicating that they are isolates of a single species, based on presently applicable species demarcation criteria [43]. PAL-DNA-A isolates were 2751–2753 nucleotides in length (Table 3), containing seven ORFs including two ORFs, AV1 (776 nt) and AV2 (359 nt), in viral sense and five ORFs, AC1 (1085 nt), AC2 (407 nt), AC3 (404 nt), AC4 (143 nt), and AC5 (767 nt) in complementary sense (Figure 3). A common region (CR) was also identified in the intergenic regions of both genomes. A predicted stem-loop region containing the sequence TAATATTAC found in the CR was identified in all sequences (Figure 3). The iterative sequence (iteron)-TGGAGAC, which is the putative binding site of the replication initiator protein (Rep), was also found in the CR. 

Database searches conducted with the complete sequences of DNA-A and DNA-B revealed high degree of sequence identity (*i.e.*, >93%) with other WmCSV isolates, and low sequence identity with other begomoviruses (Table 4). For example, DNA-A of WmCSV-[PA:Pal:10] has the highest sequence identity (99.42%) with the WmCSV-[IL] (Table 4). The virion sense genes are highly conserved; the AV2 exhibiting 98.3%–100% amino acid (aa) sequence identity with the AV2 protein of the other WmCSV isolates. The AV1 protein (coding for coat protein, CP) showed 95.4%–100% identity to other WmCSV isolates but less than 81% identity to the CPs of other begomoviruses. The sequences of the AC1 protein (coding for Rep) showed 97.5% to 99.17% aa sequence identity to other WmCSV isolates. The AC3 protein (encoding for replication enhancer protein; REn) showed 94.78%–98.51% aa sequence identity to other WmCSV isolates, and 99.01%–100% sequence identity to WmCSV Palestinian isolates. REn is involved in regulating the viral level in host plants; mutation of REn leads to reduced virus titer, but is not essential for the replication of virus [44]. The AC2, overlaps with AC3 encodes the transcriptional activator protein (TrAP) which is involved in the activation of late genes [45]. The TrAP sequences of WmCSV-[PA:Pal:10] isolates have 98.98%–100% sequence identity, while the range is 92.5%–99% identity to other WmCSV isolates. The AC4 is a highly conserved small protein contained entirely within the Rep sequence but in a different reading frame [46]. AC3 and AC4 have been implicated in suppression of gene silencing [47]. The AC4 aa sequences of the Palestinian isolates are identical to each other and share 95.74%–100% identity with other WmCSV isolates, but less than 72% identity to the AC4 sequences of other begomoviruses in the databases.

DNA-B was 2760 nucleotides in length, containing two ORFs, including BV1 (764 nt) in viral sense and BC1 (995 nt) in complementary sense. Comparison of the complete DNA-B sequence of WmCSV-[PA:Pal:10] with other bipartite begomoviruses revealed 98.4% identity with WmCSV-[IL] and most distantly related (32.47%) to PepYLCIV-[IN]. The BV1 ORFs shared more than 94% with those of WmCSV-[LB] and WmCSV-[IL], while BC1 has 99.59% amino acid identity with both WmCSV isolates from Lebanon and Israel. A double mutation at nt 524 changed A into G thus turning the ATA codon into ATG, and at nt 533 replaced G by T thus turning the start codon ATG into ATT, these mutations moved the BV1 ORF 9 nucleotides upstream. Sequence alignment of the nine WmCSV strains available in the database show that WmCSV-[IL], and WmCSV-[LB] have the same point mutation. However, this mutation did not seem to have an effect on viral pathogenicity as was reported by Abudy *et al.* [6]. Overall sequences of DNA-B from Palestine and Israel have high levels of sequence identity, suggesting that the WmCSV present in Palestine was introduced from Israel.

**Table 4:** Percentage nucleotide and amino acid sequence identities (%) between WmCSV-[PA:Pal:10] isolate with other WmCSV isolate sequences available in the databases.

Virus	Total nt		DNA-A														DNA-B			
			AV2		AV1		AC1		AC2		AC3		AC4		AC5		BC1		BV1	
	DNA-A	DNA-B	Nt	aa	nt	Aa	Nt	Aa	nt	Aa	Nt	Aa	nt	aa	nt	aa	nt	aa	nt	aa
WmCSV [YE]	97.60	95.80	99.44	98.32	97.68	95.35	97.88	97.50	97.55	95.00	97.28	94.78	98.61	95.74	98.57	96.86	89.86	91.56	96.60	93.65
WmCSV [SD]	98.22	95.36	98.89	98.32	98.97	**100.00**	98.25	98.61	97.30	94.17	97.78	95.52	100.00	**100.00**	99.09	97.65	89.96	91.87	95.03	92.11
WmCSV [JO]	99.20	95.80	99.72	**100.00**	99.61	**99.61**	99.45	**99.17**	98.28	95.83	98.27	96.27	100.00	**100.00**	99.61	**99.22**	90.16	91.46	96.60	92.59
WmCSV [IL]	99.42	**98.26**	100.00	**100.00**	99.49	**99.61**	99.63	**99.17**	98.77	**96.67**	98.77	**97.76**	100.00	**100.00**	99.61	**99.22**	91.57	**99.59**	97.78	**94.71**
WmCSV [IR]	98.26	94.46	99.44	99.16	98.97	**100.00**	98.16	98.06	96.57	92.50	97.04	95.52	100.00	**100.00**	99.22	97.65	89.66	91.06	96.56	92.67
WmCSV [LB]	99.06	**98.26**	100.00	**100.00**	98.58	97.29	99.45	**99.17**	98.77	**96.67**	99.01	**98.51**	100.00	**100.00**	98.83	97.25	98.69	**99.59**	98.04	**94.74**
WmCSV [OM:1]	97.86	93.16	99.44	99.16	98.46	98.84	97.61	97.50	96.57	92.50	97.78	95.52	99.31	97.87	98.96	96.86	98.16	89.84	94.77	93.72
WmCSV [OM:2]	97.79	93.77	99.44	99.16	98.46	98.84	97.43	97.23	96.53	93.33	97.78	95.52	99.31	97.87	98.96	96.86	88.96	89.02	92.96	92.15

WmCSv-DNA-A-PAL (KC462552), WmCSV-DNA-B-PAL (KC462553).

**Figure 4:**
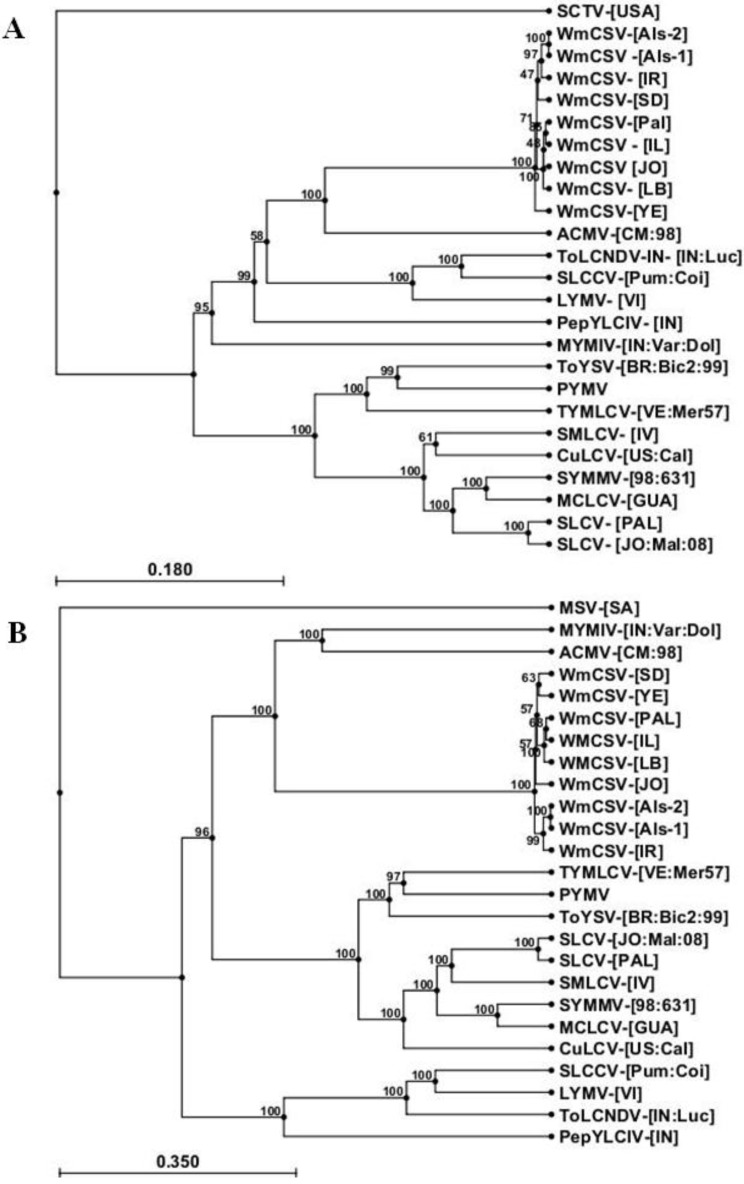
Phylogenetic trees based on a multiple sequence alignment of the complete DNA-A (**A**) and DNA-B (**B**) components of selected begomoviruses with WmCSV-[PA:Pal:10]. Trees were constructed by the UPGMA method, and branches were bootstrapped with 1000 replications. As outgroups, isolates of SCTV-[USA] and MSV-[SA] were used. Acronyms and accession numbers are presented in Table 4.

Phylogenetic trees based on the alignment of the complete nucleotide sequences of DNA-A and DNA-B of WmCSV-[PA:Pal:10] with selected begomoviruses sequences available in the GenBank database are shown in Table 5 and Figure 4. These show that WmCSV-[PA:Pal:10] clusters together with other WmCSV isolates, and the sequences segregate most closely with sequences of WmCSV isolate for Israel, suggesting its probable route of entry from Israel. WmCSV-IL has been reported to originate from Sudan [6].

**Table 5:** GenBank accession numbers of selected Begomovirus sequences used in this study for analysis WmCSV (NC_003708).

Virus Name	Abbreviation	DNA-A	DNA-B
Watermelon chlorotic stunt virus-[Yemen]	WmCSV-[YE]	AJ012081	AJ012082
Watermelon chlorotic stunt virus-[Sudan]	WmCSV-[SD]	AJ245650	AJ245651
Watermelon chlorotic stunt virus-[Oman]-Als-2	WmCSV-[Als-2]	JN618982	HE800539
Watermelon chlorotic stunt virus-[Oman]-Als-1	WmCSV-[Als-1]	JN618981	JN618980
Watermelon chlorotic stunt virus-[Lebanon]	WmCSV-[LB]	HM368371	HM368372
Watermelon chlorotic stunt virus-[Jordan]	WmCSV-[JO]	EU561237	EU561236
Watermelon chlorotic stunt virus-[Israel]	WmCSV-[IL]	EF201809.1	EF201810.1
Watermelon chlorotic stunt virus-[Iran]	WmCSV-[IR]	AJ245652	AJ245653
Tomato yellow spot virus[Brazil:Bicas 2:1999]	ToYSV-[BR:Bic2:99]	DQ336350	DQ336351
Tomato yellow margin leaf curl virus-[Venezuela]	TYMLCV-[VE:Mer57]	AY508993	AY508994
Tomato leaf curl New Delhi virus India-[India]	ToLCNDV-[INLuc]	Y16421	X89653
Squash yellow mild mottle virus-[Costa Rica:1998]	SYMMV-[98:631]	AY064391	AF440790
Squash mild leaf curl virus-[Imperial Valley]	SMLCV-[IV]	NC_004645	NC_004646
Squash leaf curl virus-[Palestine]	SLCV-[PA: Pal:10]	KC441465	KC441466
Squash leaf curl virus-[Jordan]	SLCV-[JO:Mal:08]	EF532620	EF532621
Squash leaf curl China virus -[Pumpkin:Coimbatore]	SLCCV-[Pum:Coi]	AY184487	AY184488
Potato yellow mosaic virus-[Puerto Rico]	PYMV	AY965897	AY965898
Pepper yellow leaf curl virus-[Indonesia]	PepYLCIV-[IN]	AB267834	AB267839
Mungbean yellow mosaic virus-[India]	MYMIV-[IN:Var:Dol]	AY547317	DQ061273
Melon chlorotic leaf curl virus-[Guatemala]	MCLCV-[GUA]	AF325497	AF325498
Loofa yellow mosaic virus-[Vietnam]	LYMV-[VI:02]	AF509739	AF509740
Cucurbit leaf curl virus-[California]	CuLCV-[US:Cal:00]	AF224760	AF224761
African cassava mosaic virus-[Cameroon]	ACMV-[CM:98]	AF112352	AF112353
Spinach curly top virus-[USA]	SCTV-[USA]	NC_005860	-
Maize streak virus - [South Africa: Sasri_s:2007]	MSV-[SA:Sa:07]	-	EU152254

WmCSv-DNA-A-PAL (KC462552), WmCSV-DNA-B-PAL (KC462553).

### 2.4. Discrimination between WmCSV-[PAL], WmCSV-[JO] and WmCSV-[IL] Isolates

Attempts have been made to differentiate between WmCSV-[PAL], WmCSV-[JO], and WmCSV-[IL] isolates by sequence analysis of full-length DNA-A and DNA-B genomes. Using the CLC Main Workbench program [42] to determine the *Apa* I site in the DNA-A of the three virus isolates showed no differences in the digestion profiles; the DNA-A in these isolates has 5 restriction sites yielding four DNA fragments of 1406, 1120, 138, and 88 bp. On the other hand, the DNA-B of the three virus isolates, showed that WmCSV-[JO] has 2 *Apa* I restriction sites at nt 186 and nt 1663, while WmCSV-[PAL] and WmCSV-[IL] has only 1 *Apa* I restriction site at nt 1663. A point mutation at nt 187 of WmCSV-[PAL], and WmCSV-[IL] converted Guanine (G) into Cytosine (C) which subsequently eliminated the *Apa* I site at this position. Following these findings, sequences of DNA-B of WmCSV-[LB], WmCSV-[YE], WmCSV-[SD], WmCSV-[IR], WmCSV-[OM1], and WmCSV-[OM2] available in the GenBank database were analyzed using the CLC main workbench [42]. Sequence analysis revealed that WmCSV-[LB] was shown to be similar to WmCSV-[PAL], and WmCSV-[IL], having 1 *Apa* I site at position nt 1663. However, WmCSV-[SD] also has one *Apa* I site at nt 186; a point mutation at nt 1665 converted (G) into thymine (T) and eliminated the *Apa* I site at this position. In addition, WmCSV-[YE], WmCSV-[IR], WmCSV-[OM1], and WmCSV-[OM2] were found to be similar to WmCSV-[JO], having 2 *Apa* I sites at nt 186 and nt 1663. The regions from which WmCSV-[PAL] were isolated (Jenin and Qalqilia) are on the borders with Israel. WmCSV-[PAL] isolates were found in this study to be more related to the WmCSV-[IL] isolate on both genomic or protein levels. These data suggest that WmCSV-[PAL] was introduced to cucurbit fields in Palestine from Israel either by whiteflies, or by exchange of seeds and plants between the two countries, where several viruses and viroids have been, and undoubtedly still are, disseminated worldwide through exchange of seeds having undetected infection [48].

### 2.5. Inoculation of Watermelon Plants by Particle Bombardment (Biolistic Inoculation)

Total nucleic acids extracted from symptomatic watermelon plants were used to inoculate watermelon plants, after RCA amplification, with a particle inflow gun. Severe leaf curling was observed in 24 out of 30 plants 3 weeks post inoculation (WPI) and characteristic leaf mottling and yellowing developed 6 WPI. The expected sizes of viral DNA-A (2751 bp) and DNA-B (2760 bp) could be amplified from symptomatic plants by PCR. Particle bombardment of watermelon plants with RCA-amplified viral DNA-A and DNA-B resulted in severe disease symptoms including vein yellowing and mottling. Several studies described particle bombardment (biolistic inoculation) as an efficient technique for inoculating plants with viral nucleic acids. Our results showed that 80% of inoculated watermelon plants developed disease symptoms; however, the Jordanian results showed that only 20% of inoculated watermelon plants could develop disease symptoms [16]. The efficiency of plant inoculation using the particle inflow gun was affected by several factors including plant age, gas pressure, the distance between the particle inflow gun and the plant as well as the source of the nucleic acid used for inoculation [6,49,50,51]. In this study the direct bombardment of plants with RCA products has shown to be a convenient and quicker tool for biolistic inoculation, in agreement with previous studies that successfully used RCA products for biolistic inoculation of geminiviruses [27,28].

The identification of WmCSV in Palestine which has wide agricultural trading ties with neighboring Arab countries including Syria, Iraq, and Egypt, adds to the worry that the virus may spread outwards to these countries and the rest of the world. In the Mediterranean region, WmCSV has already been detected in Israel, Jordan, and Lebanon. Hence, it is likely that given time, the virus will spread to cucurbit crops into other countries in the region. Thus, the virus poses danger to watermelon and other cucurbit crops around the Mediterranean.

## 3. Experimental

### 3.1. Samples Collection

In the summers of 2010 and 2011, disease symptoms which resemble those caused by WmCSV were observed on cucurbit plants grown in watermelon and cucumber fields located in the northern part of Palestine (Jenin, Tulkarm, and Qalqilia). Infected plants showed symptoms like yellow veins, mottling, severe stunting of young leaves, and drastically reduced fruit yield. In addition, a high population of whiteflies (*Bemisia tabaci*) was also observed in these fields. To identify the etiology of the disease, a total of 417 leaf samples were collected from symptomatic watermelon and cucumber plants. In the spring of 2013 another 84 watermelon leaf samples were collected from 7 watermelon fields in Jericho area. Samples were either processed immediately or stored at −80 °C for further analysis.

### 3.2. Nucleic Acid Extraction

Total nucleic acids were extracted from collected leaf samples as described by Dellaporta *et al.* [52]. In brief, 50 mg of leaf sample was gound into 1 mL of extraction buffer (50 mM EDTA, 100 mM Tris-HCl; pH 8.0, 500 mM NaCl, 10 mM β-mercaptoethanol). One percent SDS was added and the mixture was incubated at 65 °C for 10 min. After adding one-fifth volume of potassium acetate (5 M, pH 8.0), the mixture was kept on ice for 10 min and then clarified by centrifugation at 14,000× *g* for 10 min. An equal volume of phenol/chloroform/isoamyl alcohol (25:24:1) was added to the supernatant, and centrifuged at 14,000× *g* for 10 min. Two volumes of cold ethanol 99% were added to the supernatant and the mixture was incubated for 10 min at −20 °C. After centrifugation for 10 min at 10,000× *g*, the pellet was washed with 70% ethanol and re-suspended in sterile deionized water.

### 3.3. Detection of WmCSV-[PAL] by Polymerase Chain Reaction Using Degenerate Primers

The degenerate primer pairs PAL1v1978/PAR1c496 and PBL1v2040/PCRc1 [19] were used to detect WmCSV in leaf samples collected from symptomatic cucumber and watermelon plants. These primers amplify fragments of DNA-A (~1.1 Kb) and DNA-B (0.5 Kb) of whitefly-transmitted geminiviruses, respectively [19]. Nucleic acids obtained from healthy plants were used as negative control. PCR reactions were performed in Techne TC-Plus Thermal Cycler (Bibby Scientific Limited, Staffordshire, UK). Each reaction contained 50 ng of sample DNA, 0.25 mM dNTPs, 0.25 mM MgCl_2_, 2.5 µM of each primer, 0.5 U of Taq DNA Polymerase and 1× enzyme buffer in 25 µL final volume. The amplification program started with an initial denaturing step at 94 °C for 3 min, followed by 35 cycles of 94 °C for 1 min, 55 °C for 40 s and 72 °C for 1 min, and a final extension at 72 °C for 10 min. Amplified PCR products were resolved on 1% agarose gels stained with ethidium bromide (0.5 mg/mL).

#### 3.3.1. Rolling Circle Amplification (RCA)

Total nucleic acids were extracted from symptomatic watermelon and cucumber plants as mentioned above and subjected to TempliPhi amplification essentially according to the manufacturer’s instructions (Amersham Biosciences, Piscataway, NJ, USA). Briefly, 2 µL (10 ng to 20 ng) of total DNA were dissolved in 5 µL of sample buffer, denatured for 3 min at 95 °C and cooled down on ice for 1 min followed by addition of 5 µL reaction buffer and 0.2 µL enzyme mix. Amplification was performed for 16–20 h at 30 °C and the reaction was stopped by heating for 10 min at 65 °C to inactivate the enzyme. The amplified DNA was used as a template for the PCR to amplify the full length genome of WmCSV-[PAL].

#### 3.3.2. Restriction Fragment Length Polymorphism (RFLP)

The RFLP analysis was performed by digesting 1 μL (~300 ng DNA) of the RCA product with the restriction enzyme *Hpa* II (New England Biolabs, Frankfurt, Germany). The reaction was carried out at 37 °C for 2 h, followed by treatment for 20 min at 65 °C for enzyme inactivation, according to supplier’s instructions. DNA fragments were separated on 2% agarose gels following standard protocols [53] and visualized by ethidium bromide staining. Molecular weight marker with known reference fragments was used for the estimation of fragment sizes.

#### 3.3.3. Amplification of DNA-A and DNA-B of WmCSV-[PA]

The DNA-A and DNA-B of WmCSV-[PA:Pal:10] were amplified by PCR using the primer pairs (WmA150F/WmA1350R and WmA170R/WmA1325F) for DNA-A and (WmB672F/WmB2000R and WmB1977F/WmB695R) for DNA-B (Table 2). The PCR reaction contained 1 µL of RCA product, 0.25 mM dNTPs, 0.25 mM MgCl_2_, 2.5 µM of each primer, 0.5 U of HotStar Taq *Plus* DNA Polymerase (Qiagen, Venlo, Limburg, Netherlands) and 1x enzyme buffer in a total volume of 25 µL. Viral DNAs were amplified in a Techne TC-Plus Thermal Cycler (Bibby Scientific Limited, Staffordshire, UK) by 1 cycle of melting at 94 °C for 3 min followed by 35 cycles of melting, annealing and DNA extension conditions of 1 min at 94 °C, 1 min at 57 °C (for DNA-A primers) and 65 °C (for DNA-B primers), 2 min at 72 °C. For the last cycle, the extension time was increased to 10 min. Amplified DNA fragments were electrophoresed in 1% agarose gel in TAE buffer (Tris-Acetate EDTA) and visualized using UV transilluminator after staining in ethidium bromide.

### 3.4. Cloning and Sequence Analysis

The amplified fragments for DNA-A and DNA-B were immediately ligated into pTZ57R/T Cloning vector (Thermo Scientific, Waltham, MA, USA) and cloned according to the manufacturer’s instructions. After transformation into the JM109 strain of *E. coli*, white colonies were screened for the gene of interest by PCR and restriction digestion with *Hin*d II and *Xba* I. Clones of DNA-A and DNA-B fragments were sequenced in both directions using the following primers: M13/pUC #SO100 and M13/pUC #SO101 (Macrogen Inc., Seoul, Korea). Twenty clones of DNA-A-[PAL] and one clone of WmCSV-B-[PAL] were subjected to sequencing. Complete sequences of 25 geminiviruses infecting cucurbit crops (Table 5) were retrieved from the GenBank [54] and used for comparison using CLCmainworkbench [42] ORF finder [54] was used to identify the ORFs of DNA-A and DNA-B. After multiple sequence alignments, phylogenetic trees have been constructed using the UPGMA algorithm of CLC workbench [42]. A consensus dendrogram was generated using bootstrap value of 1000 replicates for these algorithms.

### 3.5. Discrimination between WmCSV-[PAL] and WmCSV-[IL] and WmCSV-[JO] Isolates Using in Silico Enzymatic Digestion

To discriminate between WmCSV-[PAL], WmCSV-[JO], and WmCSV-[IL] *in silico* digestion of the retrieved sequences of the three virus isolates with *Apa* I restriction enzyme was carried out using Clone manager 7, Sci-Ed software [55]. Sequences of the three isolates were used to determine the restriction site of the *Apa* I enzyme in DNA-A and DNA-B.

### 3.6. Inoculation of Plants by Particle Bombardment (Biolistic Inoculation)

The circular genomic DNA-A and DNA-B of WmCSV-[PAL] were amplified from watermenlon infected plants by RCA as mentioned above and used to inoculate 30 watermelon plants using a particle inflow gun, without vacuum, as previously described [6]. Following inoculation, plants were kept in the greenhouse and monitored for disease development. Approximately 4 WPI, all plants were tested for virus infection by visual inspection of disease symptoms and by PCR.

## 4. Conclusions

The sequence of WmCSV from Palestine shows the highest levels of identity to a WmCSV isolate from Israel, which indicates that the virus was likely introduced from Israel. Watermelon, squash, and cucumber are important crops in Palestine, which are increasingly affected by the virus, and endangering the production of these crops in the area. Geminiviruses, including WmCSV and SLCV, are widely distributed in the Eastern Mediterranean countries, thus particular attention should be paid to these viruses. Otherwise, they could invade and cause remarkable yield losses to other crops, especially with the wide distribution of the endemic population of the whitefly vector in the region. WmCSV was found to be restricted to some regions in Palestine in this study, thus, intensive efforts should be made to avoid further spread of the disease to other cucurbit-growing regions like Jericho and Tulkarm, and especially during the spring growing season where the population of the insect vector is usually high. Furthermore, collaborative efforts, between scientists in the region, should be initiated to search for resistance sources against WmCSV and SLCV in wild cucurbit species.
